# Prevention of Buttock Claudication by Preserving Antegrade Bilateral Superior Gluteal Arterial Blood Flow in EVAR for Aorto-Iliac Aneurysm Accompanied by Bilateral Internal Iliac Artery Aneurysms

**DOI:** 10.3400/avd.cr.21-00107

**Published:** 2022-03-25

**Authors:** Yuta Tajima, Hitoshi Goto, Daijiro Akamatsu, Fukashi Serizawa, Shunya Suzuki, Shinichiro Horii, Norinobu Ogasawara, Hirokazu Takahashi, Yohei Nagaoka, Takashi Kamei

**Affiliations:** 1 Department of Surgery, Tohoku University Hospital, Sendai, Miyagi, Japan

**Keywords:** EVAR for internal iliac aneurysm, buttock claudication, IBE

## Abstract

Buttock claudication (BC) is a complication of surgery for aorto-iliac aneurysms (AIAs) caused by sacrificing blood flow in the internal iliac artery (IIA). However, the preservation of antegrade blood flow of IIAs is often challenging when performing both open surgery and endovascular aneurysm repair (EVAR) for AIAs accompanied by IIA aneurysms. We performed EVAR and successfully preserved the antegrade blood flow of bilateral superior gluteal arteries using the GORE EXCLUDER iliac branch endoprosthesis with the VIABAHN endograft. BC did not occur, both subjectively and objectively, after surgery. This approach can be minimally invasive yet an effective procedure to prevent BC.

## Introduction

Buttock claudication (BC) is a complication that occurs after surgery to repair aorto-iliac aneurysms (AIAs) caused by sacrificing blood flow in the internal iliac artery (IIA).^1,2)^ However, the preservation of antegrade blood flow of IIAs is often difficult when performing open surgery and endovascular aneurysm repair (EVAR), as AIAs are accompanied by large internal iliac artery aneurysms (IIAAs), and consequently, the possibility of BC increases, especially for bilateral IIAAs.^3)^ Herein, we report a case of AIAs accompanied by bilateral IIAAs wherein postoperative BC was successfully prevented by performing EVAR while preserving the antegrade blood flow of bilateral superior gluteal arteries (SGAs) using the GORE EXCLUDER iliac branch endoprosthesis (IBE; W. L. Gore and Associates, Flagstaff, AZ, USA) with the VIABAHN endograft (W. L. Gore and Associates).

## Case Report

A 67-year-old man with hypertension and chronic atrial fibrillation taking oral direct oral anticoagulants was referred to our hospital for AIA surgery. He was an active person who enjoyed skiing and walking.

**Figure 1:**
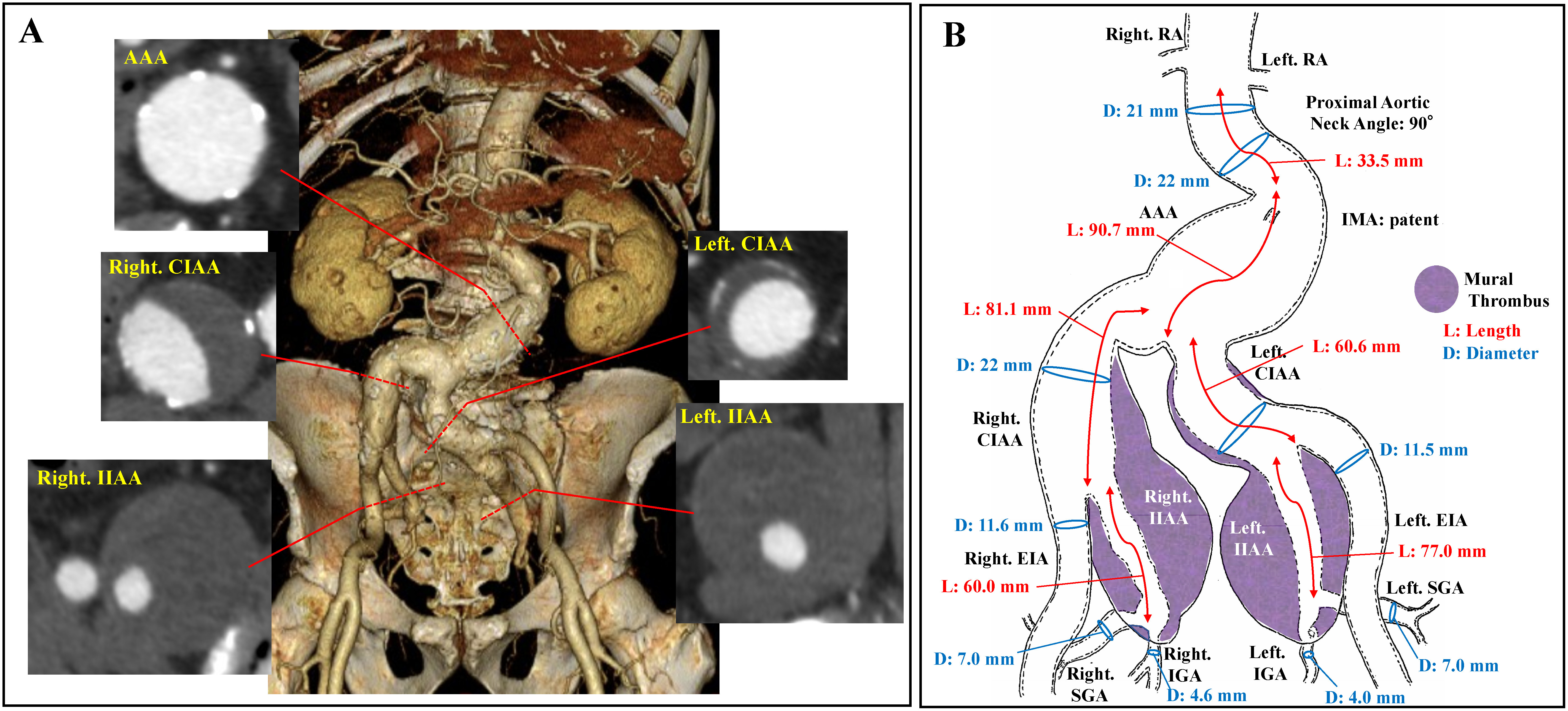
Fig. 1 Preoperative images. (**A**) Volume-rendering image and cross-sectional view of each aneurysm in enhanced computed tomography. (**B**) Schema of sizing for endovascular aneurysm repair.

AAA: abdominal aortic aneurysm; CIAA: common iliac artery aneurysm; IIAA: internal iliac artery aneurysm; IMA: inferior mesenteric artery; SGA: superior gluteal artery; IGA: inferior gluteal artery

Computed tomography (CT) revealed a 38-mm abdominal aortic aneurysm (AAA), 37-mm right common iliac artery aneurysm (CIAA), 28-mm left CIAA, 45-mm right IIAA, and 45-mm left IIAA (**Fig. 1A**).

The proximal neck length was 33.5 mm, length of the AAA was 90.7 mm, length of the right common iliac artery (CIA) was 81.1 mm, and length of the left CIA was 60.6 mm (**Fig. 1B**). All measurements satisfied the anatomical indications of EVAR using bilateral IBE.^3)^ On both sides, the SGA and inferior gluteal artery (IGA) branched separately from the IIAA. We decided to perform EVAR and use IBE with the VIABAHN endograft to avoid BC by preserving the antegrade blood flow of bilateral SGAs (7.0 mm thick).

**Figure 2:**
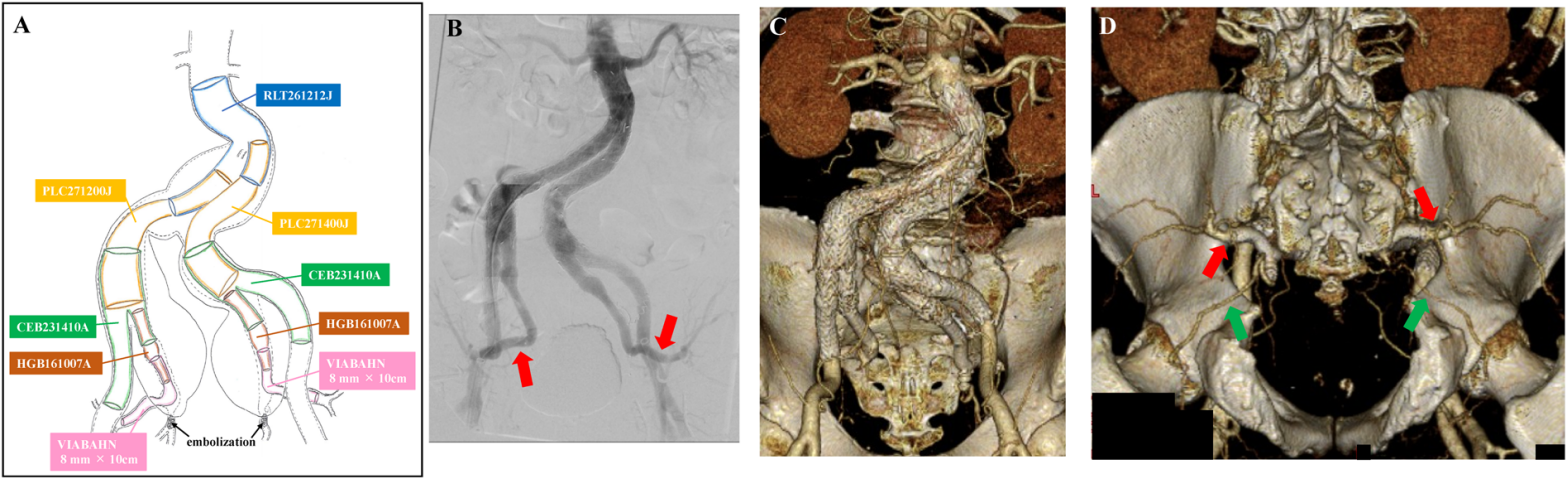
Fig. 2 Postoperative images. (**A**) Schema of the operation. (**B**) Contrast-enhanced imaging. Red arrows, good patency was observed in bilateral superior gluteal arteries. (**C**) Front view of volume-rendering image in enhanced computed tomography. (**D**) Rear view of volume-rendering image in enhanced computed tomography.

Under general anesthesia, we performed the EVAR approach via bilateral common femoral arteries by bilateral femoral incision. We placed an iliac branch component (IBC, CEB231410A) in the left CIA and embolized the iliolumbar arteries and IGA branching from the left IIAA to prevent type II endoleaks. Then, we inserted the VIABAHN endograft (self-expanding type, 8 mm×10 cm) from the left IIAA to the SGA and inserted an internal iliac component (IIC, HGB161007A) to bridge the IBC and VIABAHN endograft. Likewise, we placed an IBC (CEB231410A) in the right CIA and embolized the iliolumbar arteries and IGA branching from the right IIAA. Then, we inserted the VIABAHN endograft (self-expanding type, 8 mm×10 cm) from the right IIAA to the SGA and inserted an IIC (HGB161007A). Thereafter, we inserted the Gore Excluder C3 (W. L. Gore and Associates) main body (RLT261212J) from the right side. We inserted the contralateral leg (PLC271400J) to bridge the main body and left IBC. Finally, we inserted the contralateral leg (PLC271200J) to bridge the main body and right IBC (**Fig. 2A**). In contrast-enhanced imaging, a type II endoleak from the lumbar artery was observed in the AAA, no endoleak was observed in bilateral CIAAs or IIAAs, and good patency was observed in bilateral SGAs (**Fig. 2B**). The operation time was 422 min, fluoroscopy time was 171 min, contrast dose was 225 mL, and blood loss volume was 444 mL.

Red arrows: good patency was observed in bilateral superior gluteal arteries; green arrows: the collateral flow of inferior gluteal arteries from the deep femoral arteries

On the first day after surgery, the patient started eating and walked freely in the ward, and we added aspirin administered orally to prevent VIABAHN endograft occlusion and allow the continuation of direct oral anticoagulant therapy. Contrast-enhanced CT on day 7 after surgery revealed a type II endoleak in the AAA. However, no endoleak was observed in the CIAAs or IIAAs, and good patency was observed in bilateral SGAs (**Figs. 2C** and **2D**). On day 8 after surgery, the patient was discharged with no BC. A treadmill exercise test using near-infrared spectroscopy and a 6-min walk test were performed on the day before surgery and days 7 and 30 postoperatively (**Table 1a**). On postoperative days 7 and 30, no symptoms of BC were noted. At 6 months postoperatively, good patency of bilateral SGAs was observed, CIAAs and IIAAs slightly shrank, and AAA did not expand. The patient could enjoy skiing and walking and was highly satisfied.

**Table 1a:** Table 1a Result of the treadmill exercise test using near-infrared spectroscopy and 6-min walk test

	Treadmill with near-infrared spectroscopy (NIRO-200NX, HAMAMATSU) (speed: 2.4 km/h, tilt angle: 12%, probe position: bilateral buttocks 5 cm behind each greater trochanter)								6-min walk test on a flat surface		
	At rest		PWD	MWD					Time (s)	MWD (m)	BC
	Buttock TOI (%)			Time (s)	Distance (m)	Buttock TOI (%)/recovery time (s)*		Dissociation of ∆O_2_Hb and ∆HHb			
	Right	Left				Right	Left				
Before surgery	72	78	No pain	180	117	60/179	70/190	None	360	441	None
Day 7	69	76	No pain	180	117	59/238	70/162	None	360	459	None
Day 30	51	58	No pain	180	117	46/189	59/197	None	360	502	None

*Time from the end of walking to returning to the resting TOI. TOI: tissue oxygenation index; PWD: pain-free walking distance; MWD: maximum walking distance; O_2_Hb: oxygenated hemoglobin; HHb: deoxyhemoglobin; BC: buttock claudication

## Discussion

BC has been reported to develop on the affected side in 28% of surgeries when unilateral IIA blood flow is sacrificed.^4)^ Previous studies have also reported that the possibilities of BC on at least one side increase to ≥50%, and the rate of permanent damage increases when bilateral IIA blood flow is sacrificed.^4,5)^ These rates have been reported to increase further when the blood flow network of the SGA and IGA is interrupted.^5)^

At our institution, 20 patients with AIA, excluding ruptured cases, underwent EVAR combined with bilateral IIA embolization, a few days before EVAR, between 2016 and 2020. Although sigmoid colon ischemia and paraplegia did not occur and their performance status was not high, BC occurred on at least one side in 12 (60%) patients. Among the 20 patients, the blood flow network of the SGA and IGA was preserved on both sides in nine patients, on one side in eight patients, and sacrificed on both sides in three patients, and BC occurred in four (44%), six (75%), and two (67%) patients, respectively (**Table 1b**). In this case, the patients’ preoperative performance status was very high, and BC was predicted to highly likely occur if we had performed EVAR with bilateral IIA embolization because the blood flow network of each SGA and IGA was likely to be obstructed. Furthermore, BC was predicted to have a significant effect on the patient’s activities of daily living and quality of life, as the patient was a relatively young and highly active person. Therefore, we decided to preserve the blood flow of the IIA.

**Table 1b:** Table 1b Presence or absence of a network of the superior gluteal artery (SGA) and inferior gluteal artery (IGA) and buttock claudication after surgery in 20 patients who underwent EVAR combined with bilateral IIA embolization from 2016 to 2020 and this case

Patient	Age	Sex	Preoperative performance status	Aneurysm	Preservation of network of SGA and IGA		Buttock claudication after surgery	
					Right	Left	Right	Left
1	69	Male	2	AAA with short Bil. CIA	+	+	−	−
2	80	Male	1	AAA with short Bil. CIA	+	+	+	+
3	85	Male	2	AAA with short Bil. CIA	+	+	−	+
4	80	Male	1	AAA with short Bil. CIA	+	+	−	−
5	77	Male	2	AAA, Bil.CIAA	+	+	−	−
6	76	Male	1	AAA, short Rt. CIA, Lt. CIAA	+	+	+	+
7	80	Female	2	AAA, Bil. CIAA, Bil. IIAA	+	+	+	+
8	75	Male	2	AAA with short Lt. CIA, Rt. CIAA	+	+	−	−
9	80	Female	1	AAA, Bil. CIAA	+	+	−	−
10	83	Male	2	AAA, Bil. CIAA	−	+	+	+
11	81	Male	1	AAA, Bil. IIAA	+	−	+	+
12	76	Male	2	AAA, Rt. IIAA, Lt. CIAA	−	+	+	+
13	72	Male	1	AAA, Rt. CIAA, Lt. IIAA	+	−	−	−
14	94	Male	1	Bil. CIAA	−	+	+	+
15	87	Male	2	AAA, Bil. CIAA	+	−	+	+
16	87	Male	2	AAA with short Bil. CIA	−	+	−	−
17	67	Male	2	AAA with short Rt. CIA, Lt. IIAA	+	−	+	+
18	83	Male	1	Bil. IIAA	−	−	−	−
19	72	Male	1	Bil. CIAA, Bil. IIAA	−	−	+	+
20	80	Male	2	AAA, Bil. IIAA	−	−	+	+
**This case**	**67**	**Male**	**0**	**AAA, Bil. CIAA, Bil. IIAA**	−*	−*	−	−

*Preservation of antegrade flow of bilateral SGA. EVAR: endovasucular aneurysm repair; IIA: internal iliac artery; AAA: abdominal aortic aneurysm; Bil: bilateral; CIA: common iliac artery; CIAA: common iliac artery aneurysm; Rt: right; Lt: left; IIAA: internal iliac artery aneurysm

Methods of preserving the blood flow of the IIA in AIA surgery include reconstruction by open surgery, EVAR with external iliac artery-IIA bypass, and EVAR using an iliac branched device including IBE.^6,7)^ However, these methods are problematic for AIAs when accompanied by large IIAAs. If open surgery were to be employed in this case, reconstruction of either the SGA or IGA would have been technically demanding and invasive.^3)^ This case satisfied the anatomic requirements for the placement of IBC and IIC; therefore, we considered the antegrade blood flow preservation of the branches of IIA with IBE using the VIABAHN endograft feasible and selected minimally invasive EVAR.^8)^ We decided to use IBE and the VIABAHN endograft bilaterally because blood flow was expected to be insufficient if the antegrade blood flow of the SGA or IGA only on one side was preserved. We preserved the blood flow of the SGAs because the SGAs on both sides were thicker than that of the IGAs, and there were good networks between IGAs and deep femoral arteries. To prevent type II endoleaks into the IIAAs, bilateral IGAs and iliolumbar arteries were embolized. When the VIABAHN endograft is placed, dual antiplatelet therapy is preferable in principle; however, we prescribed single antiplatelet therapy only because the patient was receiving direct oral anticoagulants since before the surgery.

As an objective evaluation of BC, a treadmill exercise test using near-infrared spectroscopy and a 6-min walk test on a flat surface are performed.^9,10)^ However, we could not find a report that performed an objective evaluation of BC after surgery for AIA accompanied by bilateral IIAAs that preserved the blood flow of SGAs. In this case, BC was not found during the tests, and there were no decreases in the maximum walking distance and the 6-min walk distance on a flat surface. Furthermore, no BC was observed even after discharge; therefore, we concluded that deterioration of the quality of daily life and activities of daily living due to BC could be prevented.

Data from this case alone are insufficient to answer how much preservation of antegrade blood flow of bilateral SGAs contributed to the prevention of BC. We cannot rule out the possibility that BC was preventable even if the SGA was reconstructed only on one side or if IIA blood flow was not preserved bilaterally; thus, further accumulation of similar cases is desired. The long-term outcome of this procedure is unclear, and cautious follow-up, including migration of the device, endoleaks, aneurysm enlargement, obstruction of the stent-graft legs, and the VIABAHN endografts, is required. Given that this procedure requires expensive devices and complex techniques, we believe that the procedure should be used for carefully selected patients satisfying a high level of preoperative activity and some other conditions in addition to the anatomical conditions.^3,4)^ We recommended preserving the antegrade blood flow to bilateral SGAs (or IGAs) to prevent BC in cases that need embolization of bilateral SGAs and IGAs individually so that the bilateral blood flow network of the SGA and IGA is interrupted to exclude AIAs in conventional EVAR for very active patients.

## Conclusion

EVAR for AIA accompanied by bilateral IIAAs using IBE with the VIABAHN endograft to preserve antegrade blood flow of bilateral SGAs is a minimally invasive yet effective procedure to prevent BC in selected cases.
